# Involvement of HTLV-I Tax and CREB in aneuploidy: a bioinformatics approach

**DOI:** 10.1186/1742-4690-3-43

**Published:** 2006-07-05

**Authors:** Cynthia de la Fuente, Madhur V Gupta, Zachary Klase, Katharine Strouss, Patrick Cahan, Timothy McCaffery, Anthony Galante, Patricia Soteropoulos, Anne Pumfery, Masahiro Fujii, Fatah Kashanchi

**Affiliations:** 1 The George Washington University Medical Center, Department of Biochemistry and Molecular Biology, Washington, DC 20037, USA; 2 Center for Applied Genomics, Public Health Research Institute, Newark, NJ 07103, USA; 3 The Institute for Genomic Research (TIGR), Rockville, MD 20850, USA; 4 Department of Immunotherapeutics, Niigata University School of Medicine, Asahimachi-Dori, Niigata 951-8510, Japan; 5 Department of Virology, Niigata University School of Medicine, Asahimachi-Dori, Niigata 951-8510, Japan

## Abstract

**Background:**

Adult T-cell leukemia (ATL) is a complex and multifaceted disease associated with human T-cell leukemia virus type 1 (HTLV-I) infection. Tax, the viral oncoprotein, is considered a major contributor to cell cycle deregulation in HTLV-I transformed cells by either directly disrupting cellular factors (protein-protein interactions) or altering their transcription profile. Tax transactivates these cellular promoters by interacting with transcription factors such as CREB/ATF, NF-κB, and SRF. Therefore by examining which factors upregulate a particular set of promoters we may begin to understand how Tax orchestrates leukemia development.

**Results:**

We observed that CTLL cells stably expressing wild-type Tax (CTLL/WT) exhibited aneuploidy as compared to a Tax clone deficient for CREB transactivation (CTLL/703). To better understand the contribution of Tax transactivation through the CREB/ATF pathway to the aneuploid phenotype, we performed microarray analysis comparing CTLL/WT to CTLL/703 cells. Promoter analysis of altered genes revealed that a subset of these genes contain CREB/ATF consensus sequences. While these genes had diverse functions, smaller subsets of genes were found to be involved in G2/M phase regulation, in particular kinetochore assembly. Furthermore, we confirmed the presence of CREB, Tax and RNA Polymerase II at the p97Vcp and Sgt1 promoters in vivo through chromatin immunoprecipitation in CTLL/WT cells.

**Conclusion:**

These results indicate that the development of aneuploidy in Tax-expressing cells may occur in response to an alteration in the transcription profile, in addition to direct protein interactions.

## Background

Human T-cell leukemia virus type 1 (HTLV-I) is a complex retrovirus that causes adult T-cell leukemia/lymphoma (ATLL), a CD4 lymphoproliferative disease [1,2]. While endemic in Japan, South America, Africa, part of the Middle East and the Carribean, there is an increasing prevalence of HTLV-I seropositivity world wide [1-3]. ATL develops in 2–5% of HTLV-I-infected individuals after a long latency period of about 20–30 years [4-6]. Different clinical features have resulted in the division of this disease into four clinical subtypes characterized by increasing aggressiveness: smoldering, chronic, lymphoma, and acute ATL [7].

One important marker for the risk of ATL within patients is the percentage of abnormal T lymphocytes versus normal T lymphocytes within the peripheral blood [8]. Binucleated lymphocytes or lymphocytes containing cleaved/cerebriform nuclei (also termed "flower" cells) have been observed in blood smears of HTLV-I infected individuals and in ATLL cells [7,9-12]. These cells are representative of aneuploidy or abnormal chromosomal content which develops due to aberrant mitotic divisions [13]. Since aneuploidy has been suggested to contribute to tumorigenesis, there is a growing interest in deciphering the events in late G2/mitosis phase and defects therein that lead to aneuploidy. Additionally, aneuploidy may be associated with an acquired resistance to chemotherapeutic agents such as imatinib or 5-fluorouracil [14]; therefore, therapeutics disrupting aneuploidy development may improve upon current therapies for ATLL patients.

There is also a growing body of evidence to suggest that Tax, a 40 kDa viral oncoprotein encoded by HTLV-I, controls various aspects of cell cycle check points needed for aneuploidy. In fact, we were one of the first groups to show that Tax controls the G1/S check point [15], which was later confirmed by others [16], resulting in failure of G1 checkpoint and NER deficiency [17]. For a more comprehensive review of the cell cycle and check point controls by Tax, we recommend some of the more relevant reviews published recently [18-21]. In addition to disrupting checkpoints at the G1/S resulting in continuous cellular proliferation, Tax also directly targets a number of G2 and mitotic regulators. One of the first indication of Tax's involvement in the G2 and M phases was shown by Jin and colleagues [22] who discovered that Tax binds to hsMAD1. MAD1 and MAD2 are two of several genes that are involved in the activation of the mitotic spindle checkpoint function (MSC) following chromosomal missegregation. Tax interaction hindered the binding of MAD1/MAD2 complex to kinetochores by inducing translocation of these factors from the nucleus to the cytoplasm [23]. Furthermore, recent reports have demonstrated that Tax promotes activation of the anaphase promoting complex (APC)- APC^Cdc20p ^leading to a reduction in Pds1p/securin and Clb2p/cyclin B levels in yeast, rodent and human cells [6,24]. Overall, the degradation of these critical check point proteins results in a delay or failure in mitotic entry and progression, and is accompanied by a loss of cellular viability, resulting in aberrant anaphase progression, chromosomal instability and severe DNA aneuploidy [25-27].

Concurrently, Tax has been shown to repress cellular DNA repair by binding to Chk2 [24,28] and Chk1, thus impairing kinase activities *in vitro *and *in vivo *[25]. Moreover, Tax silences cellular checkpoints, which guard against DNA structural damage and chromosomal missegregation, thereby favoring the manifestation of a mutator phenotype in cells [18]. In such cells, rapidly induced cytogenetic damage can be measured by a significant increase in the number of micronuclei (MN) in cells knocked-out for DNAPKcs [29,30]. Therefore, it is possible that Tax perturbs many dynamic complexes that coordinate the processes of cell cycle regulation and DNA repair.

Here we present evidence that cytotoxic T cells (CTLL) stably expressing wild type Tax (CTLL/WT) exhibited a higher incidence of aneuploidy when compared to a Tax clone deficient for CREB transactivation (CTLL/703) [31]. Given the role of Tax as a strong activator of both viral and cellular transcription, we address the role of Tax-dependent transcription through the CREB/ATF pathway in the possible development of aneuploidy. We performed gene expression microarray analysis comparing CTLL/WT to CTLL/703 cells. Those genes that were either up or down-regulated in CTLL/WT cells were functionally annotated using the NIH's Database for Annotation, Visualization, and Integrated Discovery (NIH-DAVID). Next, we used an online database – PromoSer – to extract promoters of annotated genes to determine which of these genes contained CREB binding sites. Finally, chromatin immunoprecipitation was used to determine if DNA binding proteins such as Tax, Pol II and CREB were present at the promoters of the few selected genes. Our results clearly indicate that Tax/CREB binds to promoters of many genes, including Sgt1 and p97 (Vcp), which have functions in spindle formation and disassembly, respectively. The consequences of their over-expression and involvement in aneuploidy will be discussed.

## Results and discussion

### Aneuploidy prevalence in CTLL/WT cells

Tax deregulates the expression of genes that encode interleukin-2 (IL-2) and the multisubunit (IL-2R alpha, IL-2R beta, and IL-2R gamma) IL-2 receptor (IL-2R) in the early phases of HTLV-I induced ATL [32]. In the later phases of ATL, cells no longer produce IL-2 but still continue to express the IL-2R [32]. Fujii and colleagues developed an IL-2 independent system where Tax was stably expressed in a mouse IL-2-dependent T-cell line, CTLL-2, and examined the growth property of these cells in the absence of IL-2 [31]. While the Tax M47 (703) mutant activates NF-κB-dependent transcription but not CRE-dependent transcription, the reverse is true for the Tax M22 mutant [31]. They also noted that in addition to Tax's role in cAMP-responsive element (CRE) and NFκB activation pathways, Tax also increases expression of mRNAs coding for various AP-1 transcription factor family members including *c-Jun*, *JunB*, *JunD*, *c-Fos *and *Fra-1*. Genes encoding AP-1 are immediate-early genes whose products play important roles in cell activation, proliferation and transformation. Thus, an alternate pathway, i.e., AP-1, may be involved in the dysregulated phenotypes of T cells expressing Tax (CTLL) or infected with HTLV-1 [33].

Initially, these mutants were used to investigate the involvement of the transcription pathways in the transformation of CTLL-2 cells by Tax. Wild type Tax expression in CTLL-2 cells resulted in IL-2 independent growth. The Tax M47 mutant still activated the NF-κB-dependent transcription, and was able to support the growth of CTLL-2 in the absence of exogenously added IL-2. Therefore, the CREB dependent activity of Tax may not be as critical for IL-2 dependent growth, but may be needed for Tax induced transformation of cells. Tax M47 induced transformation may be accomplished through deregulation of cellular oncogenes, tumor suppressor genes, or checkpoint genes for DNA damage.

Our previous work utilizing these cell lines have shown that depending on which phase of the cell cycle DNA damage occurred, two different phenotypes were observed. Using centrifugal elutriation, we were able to fractionate cells at G1, S, or G2/M based on differences in cell volume at these distinct phases [34]. These cell fractions were gamma irradiated and harvested 24 hrs later for cell cycle analysis by propidium iodide staining and FACS. We observed that the CTLL/WT cells exhibited a distinct phenotype; at G1, these cells were able to induce a G1/S checkpoint, while at S or G2/M phase, these cells apoptosed after gamma irradiation. Conversely, CTLL/703 cells continued to proliferate without any apoptosis. We believed these differences were due, at least in part, to the differing gene expression profile of these cells and the induction of DNA damage at a particular point in the cell cycle. Interestingly, it was also observed that unirradiated CTLL/WT cells displayed a higher prevalence of aneuploidy than CTLL/703. The appearance of aneuploidy occurred in the later fractions (G2/M phase) which represented the largest cell populations. Consistent with our findings, previous reports have also indicated that centrifugal elutriation was capable of separating mixed populations of diploid and aneuploid cells [35,36].

**Figure 1:**
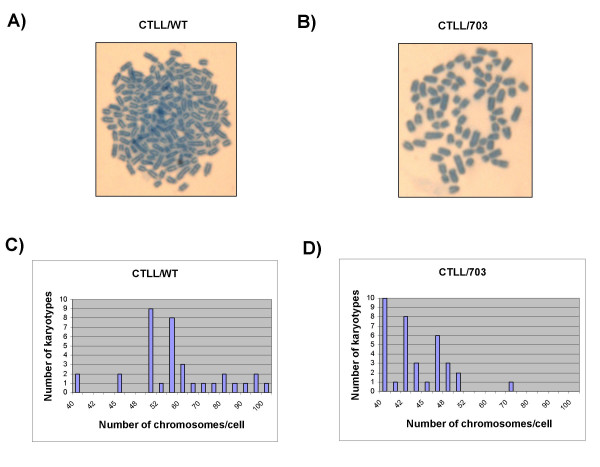
**Aneuploidy in CTLL/WT cells**. CTLL/WT and CTLL/703 cells were treated with 10 μg/ml colcemid, centrifuged, and resuspended in hypotonic solution to swell the cells. Cells were then fixed and dropped onto slides. After being stained with Giemsa and dried, slides were analyzed using the Olympus BX-60 microscope. A total of 35 metaphase spreads were counted. Representative chromosome spreads of CTLL/WT (panel A) and CTLL/703 (panel B) are displayed with 100 and 42 chromosomes, respectively. Panels **C) **and **D) **are graphical representations of the raw counts from these two cell types.

To examine the chromosomal instability in CTLL/WT cells, we first performed a metaphase chromosome spread to determine the average number of chromosomes [6,37]. Both CTLL/WT and CTLL/703 cells were processed as described in the Methods section. Thirty-five cells were analyzed for each cell type. As shown in Figure 1, CTLL/WT cells displayed higher numbers of chromosomes with an average number of 61 in contrast to CTLL/703 cells, where the average number of chromosomes were 44. The basic karyotype of the *Mus musculus *species is 2N = 40 [38]. These results support our earlier observation that wild-type Tax expressing cells (CTLL/WT), as compared to the CREB deficient Tax clones (CTLL/703), had a higher incidence of aneuploidy.

Since both of these cells are transformed, *i.e*. IL-2 independent [31], differences in transformation status cannot explain the presence of aneuploidy in one cell line and not the other. While it is possible that mutation of the Tax protein, resulting in a CREB transactivation deficient Tax (CTLL/703 cells), may disrupt interactions of Tax with late cell cycle checkpoint proteins whose dysregulation contributes to aneuploidy, however, this seems to be an unlikely event. For instance, Tax interaction with hMAD1 (also known as Txbp181) appears to be dependent on the zinc finger motif located within the N-terminus of Tax [22], and not the N-terminal domain as seen in the M47 mutant. Therefore, it appears probable that Tax may contribute to aneuploidy development, at least in part, through transcriptional activation of cellular genes critical for aneuploidy. This result would not be without precedence, since the involvement of Tax in immortalization has been shown to be mediated both at the transcriptional level and by direct protein:protein interactions [18]. Furthermore, since Tax-dependent CREB transactivation was deficient in CTLL/703 and not in CTLL/WT cells (see below), it would appear that those genes involved in aneuploidy development may be CREB-dependent.

### Gene expression profiling and promoter analysis

**Figure 2:**
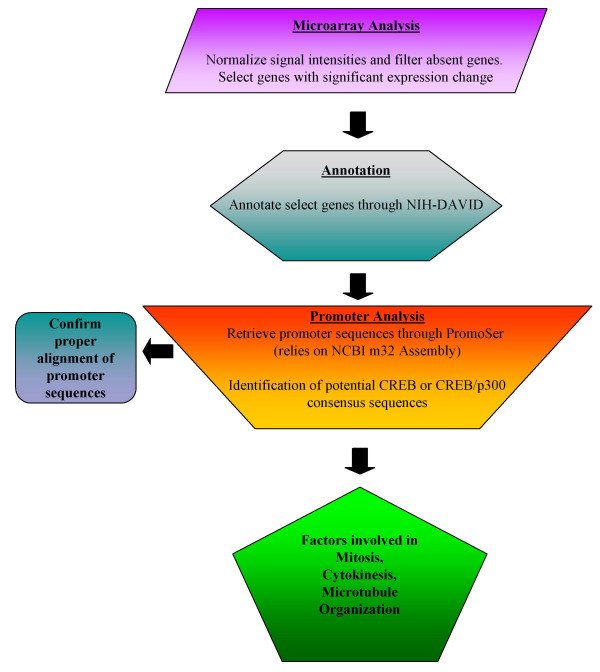
**Overview of microarray analysis, annotation, and promoter analysis**. A schematic depicting the workflow of the project. Gene expression analysis of CTLL/WT and CTLL/703 cells was performed utilizing the Affymetrix's Murine Genome U74A GeneChip. Genes that were either up-or down-regulated in CTLL/WT cells by a magnitude of at least two-fold were functionally annotated using NIH's DAVID bioinformatics program. Next, promoter sequences (2100 bp surrounding the predicted TSS) were retrieved from PromoSer. One third of the promoter sequences retrieved were checked for proper alignment against the mouse genome using Blastn and MapViewer tools through NCBI. CREB (TGACGT/C, A/GGGAGT) consensus sequences were obtained through TRANSFAC database and searched within the promoters obtained. Factors that contained the CREB sequences within their promoters were further probed for genes that contribute directly to mitosis, cytokinesis, and microtubule organization.

To begin to examine the contribution of Tax/CREB-dependent transcription in aneuploidy development, we compared the transcription profiles of CTLL/WT and CTLL/703 cells by microarray analysis. Our method for comparing the contribution of Tax/CREB to the aneuploidy phenotype is depicted in Figure 2. Through this analysis we obtained a global expression profile of the wild-type Tax-expressing cells as compared to Tax-703 expressing cells and subsequently narrowed our list by determining which genes contained CREB-response elements and potential aneuploidy associated genes. Microarray analysis was performed utilizing cytoplasmic mRNA from both cells and the Affymetrix Murine Genome U74A Array. Analysis was performed in duplicate to minimize inter-chip variability. After normalizing the fluorescence intensity of each probe and filtering for differentially expressed genes with a difference of at least 2-fold across experiments, a gene list was compiled. These genes were then functionally annotated utilizing the Database for Annotation, Visualization, and Integrated Discovery program (DAVID) [39] to generate a list of 439 genes differentially regulated in CTLL/WT cells [Additional files 1 and 2]. The majority of these differentially regulated genes were up-regulated in CTLL/WT cells (412 genes increased out of a total of 439 annotated genes, 94%) and many have been shown to be up-regulated in wild-type, and not in M47, Tax-expressing cells, including p21/waf1 [40], cyclin D2 [15], and Jun B [41], suggesting a correlation between our gene expression profile and previously published results.

Recent studies have utilized microarray analysis to determine whether gene sets were under the control of similar transcription factors by analyzing the promoter sequences for transcription factor binding sites [42-46]. We hypothesized that those genes differentially regulated in CTLL/WT cells would probably be CREB/ATF-dependent either directly or indirectly. This is based on the assumption that CTLL/703 cells contain mutated Tax, which is unable to transactivate viral or cellular transcription through the CREB/ATF pathway [31]. To determine which promoters were in fact directly CREB/ATF-dependent, we extracted promoter sequences of the differentially expressed mouse genes utilizing PromoSer, a web-based promoter database extraction tool [47,48]. This retrieval database utilizes the mm4 draft release of the mouse genome. Promoter sequences encompassing 2000 bp upstream and 100 bp downstream of the predicted transcription start site (TSS) were retrieved. Of the 439 differentially regulated genes, only 341 promoter sequences could be extracted from the database (77% recovery) under the conditions used. One third of the promoters retrieved were initially compared against the mouse genome using NCBI's Blastn to verify that these sequences were properly aligned. Next, promoter sequences were searched for CREB consensus sequences (obtained through the Transfac database and literature searches [49-52]) to determine which promoters might be CREB-responsive. Interestingly, 28% of these promoters (95 out of 341 extracted promoter sequences) were found to contain CREB binding sites. These annotated genes with corresponding fold change are depicted in Table 1. Most of the genes identified are involved in a number of pathways including transportation, signaling, cell cycle, transcription and RNA processing, metabolism, stress response, and cytoskeletal protein binding. To determine whether CREB-dependent promoters were preferentially activated, we also performed searches for NFκB and SRF response elements within the extracted promoters. Both of these transcription factors have been shown to be responsive to Tax [53]. Of these promoters, only 2% (7 out of 341 promoters) were found to contain NFκB recognition sequences and less than 1% (2 out of 341 promoters) contained SRF sequences (data not shown). Therefore, there was a selective preference in Tax expressing cells to activate CREB-dependent promoters. Through a comprehensive PubMed and DAVID search, we determined which genes were involved in mitosis/cytokinesis and thus were likely candidates to contribute to the development of aneuploidy Table (1).

**Table 1:** Cellular genes containing CREB response element activated by Tax

**Genbank Accession Number**		**Gene**	**Fold Change**	**Transcription/RNA Processing**	**Peptidase**	**Cytoskeletal Protein Binding**	**Transport**	**M etabolism**	**Transm em brane Receptor**	**Signaling**	**Cell Cycle**	**Stress Response**
AF064071	Apaf1	a po pto tic protease activating factor 1	2.5		**X**							
D87898	Arf1	ADP-ribosylation factor 1	2.6				**X**			**X**		
BC010700	Atp5c1	ATP synthase, H+ transporting, m itochondrial F1 com plex, gam m a polypeptide 1	2.1				**X**					
NM_009729	Atp6v0c	ATPase, H+ transporting, V0 subunit C	2.3				**X**					
AF183960	Ccrn4l	CCR4 carbon catabolite repression 4-like (S. cerevisiae)	2.1								**X**	
BC054097	Cetn3	centrin 3	2.2								**X**	
AF016583	Chek1	checkpoint kinase 1 hom olog (S. pom be)	2.3							**X**	**X**	
NM_009942	Cox5b	cytochrom e c oxidase, subunit Vb	2.3				**X**					
BC010197	Cpe	carboxypeptidase E	17.3		**X**							
U73445	Dld	dihydrolipoam ide dehydrogenase	2.4				**X**					
NM_007897	Ebf1	early B-cell factor 1	2.9	**X**								
D43689	Fdx1	ferredoxin 1	4.3				**X**					
AJ534939	Sm c2l1	SM C2 structural m aintenance of chrom osom es 2-like 1 (yeast)	2.8								**X**	
AB093214	Lpin1	lipin 1	2.4					**X**				
NM_010239	Fth	ferritin heavy chain	2.4				**X**					
AF024620	Gabrr1	gam m a-am inobutyric acid (GABA-C) receptor, subunit rho 1	2.3				**X**		**X**	**X**		
M63801	Gja1	gap junction m em brane channel protein alpha 1	7.4									
NM_010306	Gnai3	guanine nucleotide binding protein, alpha inhibiting 3	2.0							**X**		
BC005683	Grcc10	gene rich cluster, C10 gene	2.2							**X**		
NM_022310	Hspa5	heat shock 70kD protein 5 (glucose-regulated protein)	3.0									**X**
U53514	Guk1	guanylate kinase 1	4.2					**X**				
M 21931	H2-Aa	histocom patibility 2, class II antigen A, alpha	3.8						**X**			
BC010322	H2-Ab1	histocom patibility 2, class II antigen A, beta 1	3.1						**X**			
M 58595	H2-D1	histocom patibility 2, D region locus 1	2.7						**X**			
U35330	H2-DM b1	histocom patibility 2, class II, locus M b1	2.5						**X**			
NM_013820	Hk2	hexokinase 2	2.5					**X**				
BC052727	Hspa9a	heat shock protein, A	3.3									**X**
M 59821	Ier2	im m ediate early response 2	2.9									**X**
J03236	Junb	Jun-B oncogene	2.0	**X**							**X**	
NM_010724	Psm b8	proteosom e (prosom e, m acropain) subunit, beta type 8 (large m ultifunctional protea	2.6		**X**							
M16229	M dh2	m alate dehydrogenase 2, NAD (m itochondrial)	3.4					**X**				
AY176058	Nfkbib	nuclear factor of kappa light chain gene enhancer in B-cells inhibitor, beta	2.6									
AF026124	Pld3	phospholipase D3	2.4					**X**				
BC046832	Ppp1cb	protein phosphatase 1, catalytic subunit, beta isoform	2.5					**X**			**X**	
NM_011186	Psm b5	proteasom e (prosom e, m acropain) subunit, beta type 5	2.3		**X**							
D87911	Psm e3	proteasom e (prosom e, m acropain) 28 subunit, 3	2.2		**X**							
X61940	Dusp1	dual specificity phosphatase 1	8.1								**X**	
NM_008989	Pura	purine rich elem ent binding protein A	2.4	**X**								
BC026915	Rab6	RAB6, m em ber RAS oncogene fam ily	2.4				**X**			**X**		
U67187	Rgs2	regulator of G-protein signaling 2	2.5							**X**	**X**	
AF065924	Ccl1	chem okine (C-C m otif) ligand 1	0.3							**X**		
X84037	Glg1	golgi apparatus protein 1	2.4						**X**			
BC034674	Slc31a1	solute carrier fam ily 31, m em ber 1	2.9				**X**					
BC021537	Srp14	signal recognition particle 14	2.5	**X**								
BC028507	Tnfrsf9	tum or necrosis factor receptor superfam ily, m em ber 9	4.2							**X**		
AF159593	Plscr1	phospholipid scram blase 1	5.2									**X**
AF033353	Ubl1	ubiquitin-like 1	2.3		**X**							
AY162409	Vwf	Von W illebrand factor hom olog	2.3							**X**		
AF077002	Ywhah	tyrosine 3-m onooxygenase/tryptophan 5-m onooxygenase activation protein, eta po	2.0				**X**					
U77667	Zap70	zeta-chain (TCR) associated protein kinase	2.6							**X**		
BC005589	Cfdp	craniofacial developm ent protein 1	2.1								**X**	
BC008265	Psm b2	proteasom e (prosom e, m acropain) subunit, beta type 2	2.3		**X**							
AF132726	Casp8ap2	caspase 8 associated protein 2	2.3		**X**							
AF123312	H2afy	H2A histone fam ily, m em ber Y	2.1	**X**								
BC012241	Atp5o	ATP synthase, H+ transporting, m itochondrial F1 com plex, O subunit	2.5				**X**					
BC014798	Tax1bp1	Tax1 (hum an T-cell leukem ia virus type I) binding protein 1	2.3							**X**		
AF406651	Hnrpa2b1	heterogeneous nuclear ribonucleoprotein A2/B1	2.5	**X**								
AF098508	Dctn3	dynactin 3	2.4			**X**						
AF133818	Zfp265	zinc finger protein 265	2.1	**X**								
BC037732	Rragc	Ras-related GTP binding C	2.7				**X**					
AF148447	Uchl5	ubiquitin carboxyl-term inal esterase L5	2.3		**X**							
AB025406	Dstn	destrin	2.6			**X**						
BC002126	Gabarap	gam m a-am inobutyric acid receptor associated protein	2.1			**X**	**X**		**X**	**X**		
BC018430	Asna1	arsA (bacterial) arsenite transporter, ATP-binding, hom olog 1	2.2				**X**					
AB015652	Park7	Parkinson disease (autosom al recessive, early onset) 7	2.1	**X**								
BC022751	Isg20	interferon-stim ulated protein	4.8									**X**
BC005469	Vps35	vacuolar protein sorting 35	2.3				**X**					
BC005620	Cyc1	cytochrom e c-1	2.2				**X**					
BC024355	Usm g5	upregulated during skeletal m uscle growth 5	2.2									
BC051934	Sdhb	succinate dehydrogenase com plex, subunit B, iron sulfur (Ip)	2.5				**X**	**X**				
BC009167	Sugt1	SGT1, suppressor of G2 allele of SKP1 (S. cerevisiae)	2.3					**X**				
BC013510	Ndufb2	NADH dehydrogenase (ubiquinone) 1 beta subcom plex, 2	2.3					**X**				
AF276965	Ubap2	ubiquitin-associated protein 2	2.1		**X**							
BC003843	St13	suppression of tum origenicity 13	2.1								**X**	
NM_146087	Csnk1a1	casein kinase 1, alpha 1	2.5							**X**		
BC040794	Klf7	Kruppel-like factor 7 (ubiquitous)	2.7	**X**								
BC020132	Rars	arginyl-tRNA synthetase	2.7	**X**								
BC058078	Ppp1r15b	protein phosphatase 1, regulatory (inhibitor) subunit 15b	2.0	**X**								
M19279	Gus	beta-glucuronidase	2.6					**X**				
AF356006	Atp6v0b	ATPase, H+ transporting, V0 subunit B	2.5				**X**					
BC004045	Lactb2	lactam ase, beta 2	2.0					**X**				
BC021510	Appbp1	am yloid beta precursor protein binding protein 1	2.1								**X**	
BC026611	Aars	alanyl-tRNA synthetase	2.3	**X**								
BC024857	Eif2c2	eukaryotic translation initiation factor 2C, 2	2.6	**X**								
NM_009503	p97(Vcp)	valosin containing protein	3.0				**X**					
AF353669	Flnb	filam in, beta	31.9			**X**						
9 Unknowns: BC021353, BC037019, NM_026452, BC016544, AL023058, BC004013, BC032165BC032165, BC030844, and BC027100												

Several of the candidate genes that may contribute to the development of aneuploidy (Table 2) were found to be associated with and/or regulate kinetochore assembly, including dynactin 3 (Dctn3), protein phosphatase 1, catalytic subunit beta isoform (Ppp1cb), suppressor of G2 allele of SKP1 (Sugt1 or Sgt1), and ZW10 interactor (Zwint-1). Kinetochores are multi-subunit complexes (over 70 proteins in yeast kinetochores alone) that assemble on centromeric DNA and during mitosis act as the attachment site of chromosomes to the microtubules of the mitotic spindle [54-56]. The kinetochore, in addition to engaging microtubules, promotes correct attachment and corrects errors in attachment [57]. During metaphase, the centromeres of replicated sister chromatids are oriented on the spindle apparatus. In a dynamic interaction, the kinetochore associated microtubules associate with constant oscillatory movements until all chromosomes are bi-oriented at the metaphase plate.

**Table 2:** Tax activated genes involved in aneuploidy

**Genbank Accession Number**	**Gene**		**Fold change**	**Function**
D87898	Arf1	ADP-ribosylation factor 1	2.6	Membrane vesicle formation, inactivation nec. for mitotic Golgi breakdown, chromosome segregation, and cytokinesis [95–97]
BC054097	Cetn3	centrin 3	2.2	spindle cell body duplication need for bipolarity in cytokinesis [98–103]
AF016583	Chek1	checkpoint kinase 1 homolog (S. pombe)	2.3	needed for S and G2 checkpoints after DNA damage; Tax has been shown to disrupt its function [24,25,104–106]
AF098508	Dctn3	dynactin 3	2.4	light-chain subunit of dynactin complex (p24); dynactin complex involved in vesicle movement; dynactin complex interacts with NuMA and dynein in order to tether microtubules at the spindle poles and that are essential for mitotic spindle pole assembly and stabilization; recruitment by ZW10 protein to the kinetichore [107–110]
NM_009503	p97(Vcp)	valosin containing protein	3.0	aka cdc48; needed for spindle disassembly after segregation; Golgi disassembly-assembly [111–113]
BC046832	Ppp1cb	protein phosphatase 1, catalytic subunit, beta isoform	2.5	antagonist of Aurora B kinase during mitosis, i.e. regulating the protein interface between the centromeres and the mitotic spindle; possible downstream targets include kinetochore protein Ndc10, CENP-A, which substitutes for histone H3 in centromeric nucleosomes, and inner centromere protein INCENP; mutation of PP1 or inactivation was shown to result in cytokinesis defects. [114]
NM_008989	Pura	purine rich element binding protein A	2.4	involved in DNA replication and transcription; overexpression results in G2 blockage [115,116]
AJ534939	Smc2l1	SMC2 structural maintenance of chromosomes 2-like 1 (yeast)	2.8	aka CAP-E protein, that is part of the condensin protein complex originally identified in Xenopus; protein complex is needed for chromosome condensation; CAP-E has been found to interact with DNA ligase IV (DNA double-strand break repair protein) possible interaction important for genome stability [117–120]
BC009167	Sugt1	SGT1, suppressor of G2 allele of SKP1 (S. cerevisiae)	2.3	recent study shows that SGT1 is needed for kinetechore assembly [66,76]
AF077002	Ywhah	tyrosine 3-monooxygenase/tryptophan 5-monooxygenase activation protein, eta polypeptide	2.0	binds to CDC25B in a phosphorylation independent mechanism [121,122]
BC027100	Zwint-1	ZW10 interactor	2.1	specifies the kinetochore association of ZW10 which may act as part of, or immediately downstream of, the wait anaphase tension-sensing checkpoint [123,124]

While depletion of these particular factors have been shown to cause aneuploidy, over-expression of these factors may prove to be equally problematic. There are instances where over-expression of kinetochore or associated proteins such as dynamitin (p50; [58]), CENP-H [59], and CENP-A [60] led to disruption of the dynactin or kinetochore complex. In the case of CENP-H, over-expression contributed to the appearance ofaberrant micronuclei, a sign of aneuploidy. Therefore we envision a similar scenario where over-expression of dynactin 3 (p24), whichacts as a light-chain subunit of the dynactin complex that tethers microtubules to the kinetochore and was found to bepresent stoichiometrically at one molecule per complex, leads to sequesteration of other components of the dynactin complex away from microtubules leading to aneuploidy.

### *In vivo*confirmation of CREB binding sites

Chromatin immunoprecipitation (ChIP) has been used to determine if specific proteins bind to regions of a genome *in vivo *[61], to identify transcription factor binding to promoters [62,63], and to identify the binding of modified proteins to DNA *in vivo *[64,65]. To confirm that genes regulating aneuploidy were in fact transcriptionally activated in a CREB-dependent manner, a ChIP assay was performed on a few of the Tax regulated genes in CTLL cells. Sgt1 and p97 were amongst the list of the genes identified based on the presence of potential binding sites for CREB. Sgt1 has been shown to be an essential protein and a critical assembly factor for kinetochore assembly [66]. Experiments have been carried out in the past to demonstrate the functional significance of Sgt1. RNAi mediated Sgt1 depletion in HeLa cells leads to mitotic delay due to activation of the spindle checkpoint. Sgt1 depletion also led to the reduction in kinetochore levels of three MSC components – Mad1, Mad2 and BubR1 [66].

**Figure 3:**
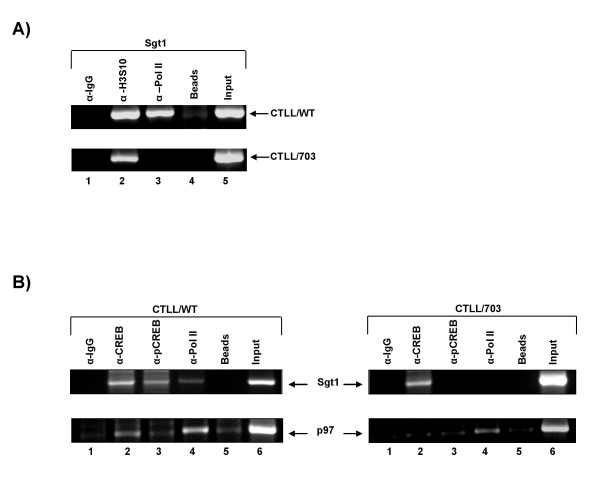
**Recruitment of CREB and basal transcription machinery at the Sgt1 and p97/Vcp promoters**. **A) **Presence of Pol II on Sgt1 promoter. ChIP analysis of Sgt1 promoter using IgG, anti- H3S10, and anti- Pol II antibodies (α-RNAP II, Santa Cruz, polyclonal rabbit #N-20)compared to input (lanes 1, 2, 3 and 5, respectively). IgG and beads only (lanes 1 and 4) were used as negative controls. Top panel corresponds to typical results from CTLL/WT cells, while the bottom panel corresponds to results from CTLL/703 cells. **B**) Presence of CREB and Pol II on Sgt1 and p97 promoters. ChIP analysis of Sgt1 and p97/Vcp promoters using antibodies against CREB, p-CREB and Pol II (lanes 2, 3 and 4). The Pol II antibody recognizes the phosphorylated elongating Pol II complex (α-Ser 2P CTD (H5, Covance)). IgG and Beads alone (lanes 1 and 5) were used as negative controls.

As shown in Figure 3A, the results from an initial ChIP assay demonstrated that RNA Polymerase II (Pol II) was present at the promoter for Sgt1 in CTLL/WT and not in CTLL/703 cells. This indicated that Pol II was not recruited in the Tax mutant CTLL/703 cells and that the Sgt1 promoter in CTLL/WT cells was transcriptionally active. Histone H3- phosphorylated serine 10 (denoted as H3S10) was used as a positive control for our ChIP assay in CTLL/WT and CTLL/703 cells. To determine whether CREB bound *in vivo *to the promoters of Sgt1 and p97/Vcp genes, we utilized CREB and phosphorylated CREB (active form of CREB, denoted as p-CREB) antibodies. Results from the ChIP assay for the promoters of Sgt1 and p97/Vcp are shown in Figure 3B. Pol II recruitment was used as a positive control. While CREB, p-CREB and Pol II were present at the Sgt1 promoter in CTLL/WT cells, we only observed the presence of CREB and not p-CREB or Pol II at this promoter in CTLL/703 cells (top right and left panels). This suggested that in CTLL/WT cells there was an active recruitment of the transcription machinery on the Sgt1 promoter. Furthermore, the presence of a phosphorylated CREB on this promoter is an indication of an active transcriptional complex [67]. We next performed similar experiments for the p97/Vcp promoter and observed the presence of CREB and Pol II at the proximal promoter region in the CTLL/WT cells. Interestingly, we observed no p-CREB binding (always similar levels to the background beads) in CTLL/WT cells. A different scenario emerged in the CTLL/703 cells, where only Pol II, and not CREB or p-CREB were present at this promoter, indicating that in the absence of Tax and CREB, there might be a paused transcriptional complex on this promoter (Figure 3B, bottom right and left panels).

**Figure 4:**
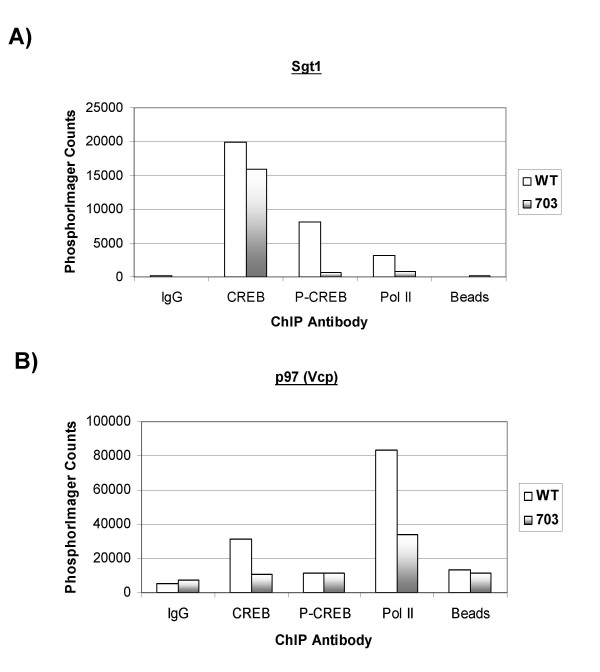
**Sgt1 and p97 ChIP results**. **A) **and **B) **are the graphical representations of the Sgt1 and p97 ChIP results obtained from CTLL/WT and CTLL/703 cells shown in Figure 3 (Average of two experiments).

A graphical representation for the same ChIP results from Figure 3 were plotted using Phosphor Imager counts (Figure 4A and 4B), where less CREB binding was observed in the CTLL/703 as compared to the CTLL/WT cells. Additionally, Pol II recruitment was found to be higher in CTLL/WT than in CTLL/703 cells. The increased recruitment of both CREB and Pol II in wild-type Tax-expressing cells, supports the hypothesis that Tax increases (either directly or indirectly) the expression of these genes.

**Figure 5:**
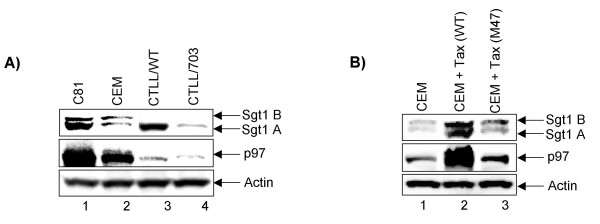
**Effect of Tax on Sgt1 and p97 protein levels**. **A) **One hundred microgram of total cell extracts from human (CEM and C81) and mouse (CTLL/WT and CTLL/703) cells were prepared and ran on a 4–20% SDS PAGE. All three C81, CTLL/WT, CTLL/703 express Tax and CEM served as a negative control. Western blots were with anti-Sgt1 (1:1000), anti-p97 (1:500) and anti-actin (1:5000) antibodies. Two forms of Sgt1 (A and B) were observed in human cells, where the A form is wild type protein and B form is the splice variant. **B**) Five microgram of either wild type or M47 Tax was transfected into CEM (5x10^6^/sample) cells. Following transfection cells were kept at 37°C for 48 hrs, followed by preparation of total extract and processed for western blot using a 4–20% SDS-PAGE. Similar antibodies as in panel A were used for the western blot and immune complexes were detected using ECL.

Finally, we performed two confirmatory assays for the up regulation of these gene products by Tax. Results in Figure 5A show western blots from two wild-type Tax expressing cells (C81 and CTLL/WT), mutant Tax expressing cells (CTLL/703), and one negative control (CEM). When examining for Sgt1 levels, we observed two forms of this protein in human cells (A and B), where the A form is the original gene product and the B form is a splice variant [68]. Interestingly, the A form was predominantly seen in both wild type Tax expressing human and mouse cells. When examining p97 levels, we observed more pronounced activation in human cells (panel A, Lanes 1 and 2) as compared to the mouse cells. Similar levels of Tax were present in both CTLL/WT and CTLL/703 cells (data not shown). We next examined the effect of Tax on endogenous Sgt1 and p97 promoters in human cells. Results from Figure 5B indicate that only wild type and not the M47 mutant was capable of activating both Sgt1 and p97 genes *in vivo*. This is critical because transfection with Sgt1 and p97 promoters may not have all the necessary elements (i.e. chromatin structure, enhancer-less promoter, proper promoter start site, and promoter distance in relation to the gene) needed to observe activated transcription by Tax *in vivo*.

### Direct effect of Tax on gene promoters

**Figure 6:**
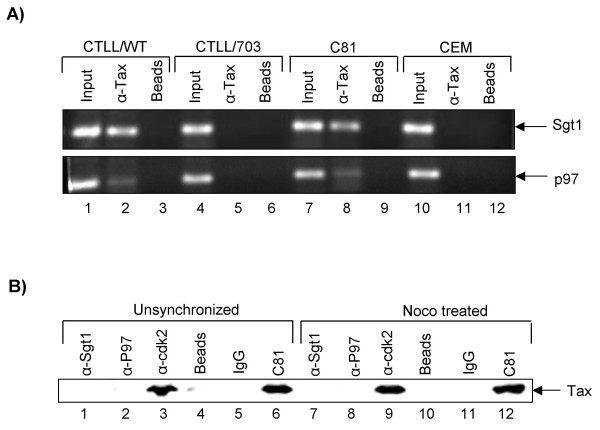
**Effect of Tax on cellular promoters and its protein-protein interaction**.**A) **Chromatin immunoprecipitation experiments were used to test whether Tax could occupy Sgt1 or p97 promoters. CTLL/WT and CTLL/703 (7.5 × 10^6 ^cells/ChIP), as well as C81 (Tax expressing, 5.5 × 10^6 ^cells/ChIP) and CEM (negative control with no Tax expression, 5.5 × 10^6 ^cells/ChIP) were cross-linked, and processed for ChIP assay. Lanes 1, 4, 7 and 10 are "input" lanes where no immunoprecipitation was performed prior to PCR (positive control). Lanes 3, 6, 9 and 12 contained no antibody, and only beads, for the immunoprecipitation (negative control). Lanes 2, 5, 8 and 11 used a mixture of 500 ng of each TAb anti-Tax monoclonal antibodies (169, 170, 171 and 172; amount of each antibody judged by running 100 ng of each purified antibody on 4–20% SDS-PAGE and stained for Heavy and light chains) as the experimental sample. The high salt wash step after immunoprecipitation contained 1000 mM (as opposed to 500 mM) salt. **B**) Two set of C81 cells were used for immunoprecipitation followed by western blot against Tax. First, unsynchronized C81 cells (2 mg total cellular extract, Lanes 1–6) where majority of cells were at the G1 phase (71%), were used for immunoprecipitation with anti-Sgt1 (1 μg), anti-p97 (1.2 μg), anti-cdk2 (0.75 μg) and IgG (1.2 μg) followed by western blot with anti-Tax polyclonal antibody. We have previously used this method to define a list of Tax binding proteins using low and high salt wash conditions [69]. We also enriched C81 cells for G2/M population (67%) by treating with low serum (1%) and nocodazole (Noco, 50 ng/ml) for 72 hrs prior to the immunoprecipitation [15]. Lanes 4, 5, 10 and 11 served as negative control for IP, and lanes 3 and 9 served as positive control for Tax binding protein under high salt conditions (cdk2). Lanes 6 and 12 were total cellular extract (Input, 50 μg) and lanes 1, 2, 7 and 8 served as the experimental sample. The high salt wash after immunoprecipitation contained 1000 mM salt.

An important question is whether Tax affects the expression level of genes listed in Tables 1 or 2 or alternatively, does Tax directly interact with any of these proteins and subsequently control aneuploidy. Although, we couldn't logistically design experiments to test all the genes listed in Tables 1 or 2 for promoter occupancy by Tax or direct protein-protein interaction, we decided to focus on the same two Sgt1 and p97 genes tested previously. We initially performed a ChIP experiment for the presence of Tax (using four TAb monoclonal antibodies) on these two promoters. Results in Figure 6A indicate that only WT, and not M47 mutant, Tax was present on both Sgt1 and p97 promoters (compare lanes 2, 5 and 8). Interestingly, we could always observe presence of more Tax on Sgt1 than p97 promoter. However, the presence of more Tax didn't correlate with increased expression levels for these two promoters (fold activation by Tax: Sgt1, 2.3 fold; p97, 3.0 fold). Next, we asked whether Tax could physically bind to either Sgt1 or p97 proteins in either an unsynchronized culture system (where the majority of cells are at the G1 phase) or cells enriched in the G2/M phase. We used human C81 cells for these immunoprecipitations followed by a high salt wash and western blot for Tax. We have previously used this stringent wash condition to identify some of the Tax binding proteins in C81 cells [69]. Results (Figure 6B) indicate that Tax could physically bind to cdk2 (positive control) and not Sgt1 or p97 proteins. It is important to note that we favor high salt washes for Tax immunoprecipitations, since low salt wash conditions may not dissociate tightly bound complexes. Collectively, results in Figure 6 indicate that Tax directly activates some of the critical proteins needed for development of aneuploidy, although with the caveat that our experimental procedures cannot fully rule out the possibility of direct protein-protein interaction between Tax and complexes that regulate aneuploidy in transformed cells.

Finally, given our results in Table 2, we wondered how over-expression of some of these genes by Tax could contribute to aneuploidy? A careful examination of the p97 and Sgt1 literature indicates that when these proteins are over-expressed, they deregulate control of few cellular pathways including apoptosis, cell division, and cell cycle. For instance, over-expression of p97 in Tax expressing cells could exhibit multiple functions including a role in anti-apoptosis and metastasis by activation of the NFκB signaling pathway [70], as well as its binding to Cdc48 and its adaptors, Ufd1-Npl4. Cdc48 and Ufd1-NPl4 regulate membrane related functions and mitotic spindle disassembly by directly binding to membrane-associated proteins or spindle assembly factors, modulating their interactions with membranes or spindles, respectively. The AAA ATPase CDC48 was first identified in *Saccharomyces cerevisiae *as a cell cycle division gene. Cdc48, as well as its homolog p97 in vertebrates, forms a homohexameric ring that functions as an active ATPase. Together with its adaptor proteins, the Ufd1-Npl4 heterodimer, the Cdc48/p97-Ufd1-Npl4 complex participates in a variety of membrane-related functions, including morphological transformations in cell division, particularly in establishing a proper G1 phase after mitosis. Consistent with this idea, Cdc48/p97-Ufd1-Npl4 is required for post-mitotic nuclear envelope reassembly [71], one of the major morphological transformations occurring at the end of cell division.

Over-expression of the Cdc48/p97-Ufd1-Npl4 complex could also assist in modification of chromatin by removing proteins involved in chromosome condensation from the highly condensed mitotic chromatin. In addition, this complex may directly regulate nuclear envelope assembly by assisting proper assembly of nuclear envelope proteins on the chromatin, which puts the proteins at the right place to coordinate post-mitotic chromosome decondensation with nuclear envelope assembly [72]. A third function of over-expressed p97 could be its role with Cdc48 and the execution of "Start" in G1 by mediating the proteolysis of the G1-CDK inhibitors i.e., Far1 [73]. This would be consistent with the idea that Cdc48/p97-Ufd1-Npl4 has a general role in regulating proper M-G1 transition [72]. Finally, over-expressed p97 ATPase could extract class I MHC from ER membranes [74]. Polyubiquitinated substrates preferentially bind by p97 ATPase in complex with two adaptor proteins, Ufd1 and Npl4. This association triggers retrotranslocation of the substrates from the ER membrane, followed by proteasome-mediated degradation [75].

Increased expression of Sgt1 induced by Tax could also affect the dynamics of its many protein partners resulting in efficient substoichiometric complexes. Sgt1 (TPR+CS domain) binds to Skp1 and this interaction might play a role in activation of multiprotein complexes CBF3 and SCF [76], assembly of the CBF3 complex, its turnover, and influence proper kinetochore function [77]. Sgt1 (TPR+CS domain) binds to Ctf13 and this binding might influence proper CBF3 assembly [78]. Sgt1 also binds Rad6, which is involved in DNA repair and protein degradation as well as cell cycle progression [79]. Furthermore, Sgt1p contributes to the activity of the cyclic AMP (cAMP) pathway and physically interacts with the adenylyl cyclase Cyr1p/Cdc35p, where a Gα subunit-type protein called Gpa2p and the GTP binding Ras proteins both of which are implicated in adenylyl cyclase activation [80-83]. The major effector of cAMP in eukaryotes is the cAMP dependent protein kinase, or protein kinase A (PKA) [84]. In the absence of cAMP, the catalytic subunit of PKA is found in an inactive complex with the regulatory subunit. The binding of cAMP by the regulatory subunit leads to dissociation of the complex and activation of the catalytic subunit [85]. In budding yeast, the adenylyl cyclase pathway is notably involved in cell growth control and stress responses [80], and also regulates the cell cycle by modulating G1 cyclin expression [86-88] and the activities of the anaphase-promoting complex/cyclosome and the SCF pathway [89].

Therefore, given the role of Sgt1 in several different types of multimeric protein complexes, it is possible that it acts as some sort of protein chaperone, assembly factor, or allosteric activator. This hypothesis is consistent with structure predictions suggesting that the Sgt1 N-terminal region is similar to the TPR regions of the Sti1/Hop co-chaperone and the central CS domain adopts a fold similar to that of the p23 co-chaperone. Sti1/Hop facilitates protein substrate transfer from Hsp70 to Hsp90, whereas p23 stimulates the ATPase activity of Hsp90 and the release of bound substrate [90]. Finally, several experimental observations are also consistent with a role for Sgt1 as a co-chaperone or assembly factor. First, Sgt1 is required to functionally activate the Skp1 and Ctf13 subunits of the CBF3 kinetochore complex but is not itself a subunit of this complex [76]. Second, Sgt1 may be present in substoichiometric quantities in SCF [76] and Cyr1 complexes regulating the M to G1 transition, and thirdly Sgt1, like SKP1, is required for both the G1/S and G2/M transitions during the cell cycle [79].

## Conclusion

The Tax oncoprotein is considered the cause for mitotic aberrations in HTLV-I infected cells. In addition to binding checkpoint factors and interfering with the MSC function, we propose a mechanism by which Tax induces these aberrations through over-expression of factors associated with kinetochore assembly. Wild-type Tax-expressing cells displayed higher incidence of aneuploidy than the CREB transactivation deficient mutant. Through microarray and promoter analyses, we have identified 95 genes that were over-expressed in CTLL/WT cells which may be regulated through CREB activity. Furthermore, 11 of these genes are involved in G2/M phase regulation, in particular kinetochore regulation. We have confirmed the regulation of Sgt1 and p97/Vcp by CREB and Tax *in vivo *by performing ChIP analysis. The over-expression of these factors may contribute to the observed aneuploidy phenotype and disrupt proper targeting of their binding partners. The mitotic aberrations generated may be in the form of improper spindle attachment and disassembly process that results in chromosomal mis-segregation and hence aneuploidy.

## Methods

### Cell culture

Chronic T Lymphocytic Leukemia (CTLL) cells stably tranfected with either wild-type Tax or the M47 Tax mutant (^319^LL → AS) have previously been described [31]. For the purpose of this study, they are designated as CTLL/WT (CTLL-2 cells transfected with wild type Tax) and CTLL/703 (CTLL-2 cells transfected with a CREB Tax mutant, M47). A comparison of Tax expression in WT and 703 cells has been published previously [15,34]. Cells were grown in RPMI-1640 with 10% FBS, 1% streptomycin, penicillin antibiotics, and 1% L-glutamine, and at 5% CO2, 37°C.

### Metaphase chromosome spread

CTLL/WT and CTLL/703 cells were incubated in 5 ml of complete media for 48 hrs. Both cultures were treated with 50 μg colcemid for 45 mins. Next, samples were centrifuged at 160 g for 10 mins. Cells were incubated at room temperature in 0.075 M KCl for 30 minutes and then fixed in 1 ml of methanol:glacial acetic acid (3:1). Cells were then centrifuged and washed again with the fixative solution for a total of three times. Finally, cells were resuspended in 250 μl of fixative and dropped onto prechilled slides at -20°C with a water vapor film formed on them just before dropping the cell suspension to induce better chromosome spread. Three to five drops of 70% glacial acetic acid was then added to remove cytoplasm. The slides were then allowed to dry overnight at room temperature. Slides were processed in Dulbecco's Phosphate Buffered Saline (D-PBS) for 1 minute, Giemsa stain working solution (2 ml Giemsa stain diluted with 25 ml D-PBS) for 10 mins, and in distilled water for 30 secs. After being dried at room temperature, cover slips were mounted and allowed to dry. Slides were then imaged using the Olympus BX-60 microscope (100x, oil immersion) and Image-Pro Plus 5.1 software. Spreads with clearly visible and distinct chromosomes were then selected and counted.

### Cytoplasmic RNA isolation

Cells were centrifuged at 4°C, 3000 rpm for 10 min., quickly washed with D-PBS without Ca2+/Mg2+, and centrifuged twice. Pelleted cells were immediately frozen at -80°C until all time points were collected. Cytoplasmic RNA was isolated utilizing the RNeasy Mini Kit (Qiagen, Valencia, CA) according to manufacturer's directions with the addition of 1 mM dithiothreitol in Buffer RLN. Isolated RNA was quantified by UV spectrophotometric analysis and 3 μg of RNA was visualized on a non-denaturing 1% agarose TAE gel for quality control.

### Expression profiling

Six micrograms of cytoplasmic RNA from each sample was used to synthesize double-stranded cDNA using the Superscript Choice System kit and T7-(dT) 24 primer (100 pmol/μL) (Invitrogen). The cDNA was purified using phenol/chloroform extraction and ethanol precipitation. The cDNA was then used to perform *in vitro *transcription using the BioArray HighYield RNA Transcript Labeling Kit (T7) (Enzo, Farmingdale, NY). The biotin-labeled cRNA was purified using the RNeasy Mini Kit (Qiagen) and quantified by spectrophotometric analysis and analyzed on a 1% agarose TAE gel. The biotin-labeled cRNA was then randomly fragmented to ~s35–200 base pairs by metal-induced hydrolysis using a fragmentation buffer according to the Affymetrix Eukaryotic Target Hybridization protocol. Two Murine Genome U74A GeneChips (Affymetrix) were used per sample. This array contained 6,000 functionally characterized sequences from the Mouse UniGene database (Build 74) and 6,000 Expressed Sequence Tag (EST) clusters. The array was washed, primed, and stained on the Affymetrix Fluidics Station 400 following manufacturer's recommendations. cRNA was detected through a primary scan with phycoerythrin-streptavidin staining and then amplified with a second stain using biotin-labeled anti-streptavidin antibody and a subsequent phycoerythrin-streptavidin stain. The emitted fluorescence was scanned using the Hewlett-Packard G2500A Gene Array Scanner, and the intensities were extracted from the chips using Microarray Suite 4.0 (MAS4.0) software. All raw chip data was scaled in MAS4.0 to 800 to normalize signal intensities for inter-array comparisons.

### Data analysis

Comparative analyses were performed by normalizing the fluorescence intensity of each probe using robust multi-array average (RMA), as previously described [91]. The mean of the signal intensities from duplicate experiments was calculated. To filter for differentially expressed genes, those genes that were absent in both experiments were excluded from further analysis. Those genes that were either up-or down-regulated in CTLL/WT cells by a magnitude of two-fold were compiled and functionally annotated utilizing the Database for Annotation, Visualization, and Integrated Discovery program (DAVID;[39]), as detailed in Figure 2.

Utilizing Genbank accession IDs, promoter sequences [2100 bp surrounding the predicted transcription start site (TSS)] for our annotated genes were retrieved from PromoSer [47,48], a large-scale database containing the promoters of human, mouse, and rat genes. The following criteria were used for the retrieval: 1) promoter sequence encompassing 2000 bp upstream and 100 bp downstream of the TSS, 2) sequences were required to contain a quality score of at least 2 and support score of 2, i.e. TSS prediction is supported by one or more mRNAs, 3) in the case of alternative promoters only the one that is best supported and 5' was included, and 4) promoters that mapped to more than one loci or did not align well were excluded. At least one third of the promoter sequences retrieved were checked for proper alignment against the mouse genome using Blastn [92] and MapViewer [93] tools through NCBI. CREB (TGACGT/C), CREB/p300 (A/GGGAGT), NFκB (GGGAA/CTTC/TCC), and SRF (CCCATATATGG) consensus sequences obtained through the TRANSFAC database [94] were searched within the promoters obtained. Factors that contained the CREB or CREB/p300 sequences within their promoters were further probed for genes that contribute directly to mitosis, cytokinesis, and microtubule organization.

### Chromatin immunoprecipitation

Cells (5 × 10^6^/immunoprecipitation) were formaldehyde cross-linked (1% final volume, 15 mins. 37°C). Nuclei, prepared by hypotonic lysis, were resuspended in lysis buffer (1% SDS, 10 mM EDTA, 50 mM Tris-HCl, pH 8.1), sonicated to reduce DNA length to 200–1000 bp, and debris was removed by centrifugation. The chromatin solution was diluted 10-fold by ChIP dilution buffer (0.01% SDS, 1.1% Triton X-100, 1.2 mM EDTA, 16.7 mM Tris-HCl, pH 8.1, 167 mM NaCl) and precleared on protein A/G agarose beads pre-adsorbed with sonicated salmon sperm DNA (10 mg/ml) and bovine serum albumin (BSA [10 mg/ml]). The solution was then centrifuged and 1 ml of the supernatant was stored separately at 4°C to be used as input. Precleared chromatin was incubated with 10 μg of antibody overnight at 4 °C, followed by immunoprecipitation with 60 μl of protein A/G-agarose beads per immunoprecipitation. Immune complexes were washed twice with Low Salt buffer (0.1%SDS, 1% Triton X-100, 2 mM EDTA, 20 mM Tris-HCl, pH 8.1), twice with High Salt buffer (0.1%SDS, 1% Triton X-100, 2 mM EDTA, 20 mM Tris-HCl, pH 8.1, 500 mM NaCl), once with LiCl (0.25 M LiCl, 1% NP40, 1% deoxycholate, 1 mM EDTA, 10 mM Tris-HCl, pH 8.1) and once with 1 M TE, pH 8.0. Cross-linking was reversed by heating at 65°C overnight in the presence of 20 μl 5 M NaCl. DNA was recovered by digestion of proteins with 50 μg/ml proteinase K followed by phenol-chloroform extraction (twice) and ethanol precipitation. Recovered DNA was resuspended in TE, pH 8.0. 10% of the recovered DNA was used for each PCR amplification (35 cycles). The primers used for PCR are as shown in Table 3.

**Table 3:** Primers used for PCR

**Accession #**	**Gene ID**	**Forward**	**Reverse**	**Size(bp)**	**Correctly mapped**
D87898	Arf1	ACCCTTGCTCGTTCTAGTGC	GGTTTCGCTCCCACAAGAT	224	Yes
AF016583	Chek1	CCACCACACTTGCTTTCCTT	GGAATCCAAATGCACAGCTT	583	Yes
AF098508	Dctn3	TTTGGGTGTACGTCCTGACA	CAGCTCCTCCACTCGAGACT	486	Yes
NM_009503	p97(Vcp)	ATTGCCTTTGTCGATTGGTC	TCGGAAGGAAAGCTGCTCTA	228	Yes
BC046832	Ppp1cb	AGCAGGGAAGGAAGGTCATT	GGCGTTCTCACCTACGAGTC	529	Yes
AJ534939	Smc2l1	CTTACAGCCGTTTGCCTAGC	CCGTTTTGAACATGGAAAGC	439	Yes
BC009167	Sgt1	AGCCGACTTAGGAAGGAAGC	GTCTCGGAGCCCACTGTAAG	325	Yes
AF077002	Ywhah	AGGTCCCCGTAGGTATGTCC	CCCAGCCCTAACGGTCTT	507	Yes

## Competing interests

The author(s) declare that they have no competing interests.

## Authors' contributions

CdlF and MG drafted the manuscript. MG performed the metaphase chromosome spread. CdlF isolated RNA and contributed to the expression profiling experiment. AG and PS performed the expression profiling protocol on all samples. PC and TM helped with the gene expression analysis. CdlF and KS performed the promoter analysis. MG and ZK performed the ChIP experiments. MF provided some of the reagents. CdlF, AP, and FK contributed to the design, coordination, and validation of the study. All authors have read and approved the manuscript.

## Supplementary Material

Additional File 1 wtminus_grtthan_703minus_annotated. Click here for file

Additional File 2 wtminus_less_703minus_annotated. Click here for file
